# Malignant transformation of primary ameloblastoma of skull: case report and review of current literature

**DOI:** 10.3389/fonc.2024.1365625

**Published:** 2024-03-21

**Authors:** Haitong Xu, Jialiang Tan, Dongxiang Fu

**Affiliations:** Department of Neurosurgery, Guangdong Sanjiu Brain Hospital, Guangzhou, China

**Keywords:** ameloblastoma, ameloblastic carcinoma, malignant transformation, stereotactic radiotherapy, planning target volume

## Abstract

**Background:**

Since 1964, there has been a scarcity of reported cases of primary ameloblastoma (AM) or ameloblastic carcinoma (AMCa) of the skull. The clinical presentation and distinctive features of this uncommon condition at specific anatomical sites remain unclear. We report a case of malignant transformation of a primary AM of the skull situated in the frontal-temporal-parietal region and highlight its similarities to other cases reported in the literature.

**Clinical presentation:**

A 53-year-old female patient presented with a 20-day history of headaches and bilateral lower limb weakness for 10 days. Physical examination revealed slow and unsteady gait. An occupying lesion was observed in the right frontal-temporal-parietal region of the skull on the Cranial imaging. A right cranial bone tumor margin expansion resection was performed. The patient’s motor functions recovered normally after surgery. Postoperative imaging examinations showed10 tumor resection. Follow-up imaging examinations showed tumor recurrence. The patient underwent resection of the recurrent tumor. Postoperative pathological analysis revealed malignant transformation of the AM.Follow-up imaging examinations showed tumor recurrence again. The patient was admitted for stereotactic radiotherapy. Follow-up imaging examinations demonstrated no evidence of tumor recurrence and subsequent chest CT revealed no signs of metastasis.

**Conclusion:**

Primary AM or AMCa of the skull is increasingly being described in the literature, but detailed reports on the malignant transformation of primary AM of the skull are lacking. The pathogenesis of this condition remains unclear. Aggressive treatment and close follow-up may be crucial for preventing disease recurrence and malignant transformation.

## 1 Introduction

Ameloblastoma (AM) is a locally invasive tumors that originate from the odontogenic epithelium, and ameloblastic carcinoma (AMCa) is a rare odontogenic malignancy that combines the histologic features of ameloblastoma with cytologic atypia (1, 2). They are exceptionally uncommon outside the jawbones and have high recurrence rates, especially in cases where the surgical removal is not sufficiently extensive (1). In rare cases, it can metastasize, most commonly to the lungs (3). Currently, there is no consensus on the optimal treatment approach, but aggressive surgical intervention and close postoperative medical follow-up are essential. Additional treatments such as radiation therapy, chemotherapy, and immunotherapy may be considered (4). Notably, primary AM malignancy in the cranial bones (frontal-temporal-parietal regions) is extremely uncommon, and detailed reports on this phenomenon are lacking. Herein, we describe a rare case of primary malignant transformation of AM in the cranial bones. The patient underwent a surgical intervention combined with stereotactic radiotherapy. During the short-term follow-up period, tumor recurrence or metastasis was not observed. We also reviewed current literature through PubMed using the keywords.

## 2 Clinical presentation

### 2.1 Report of a case

**Figure 1:**
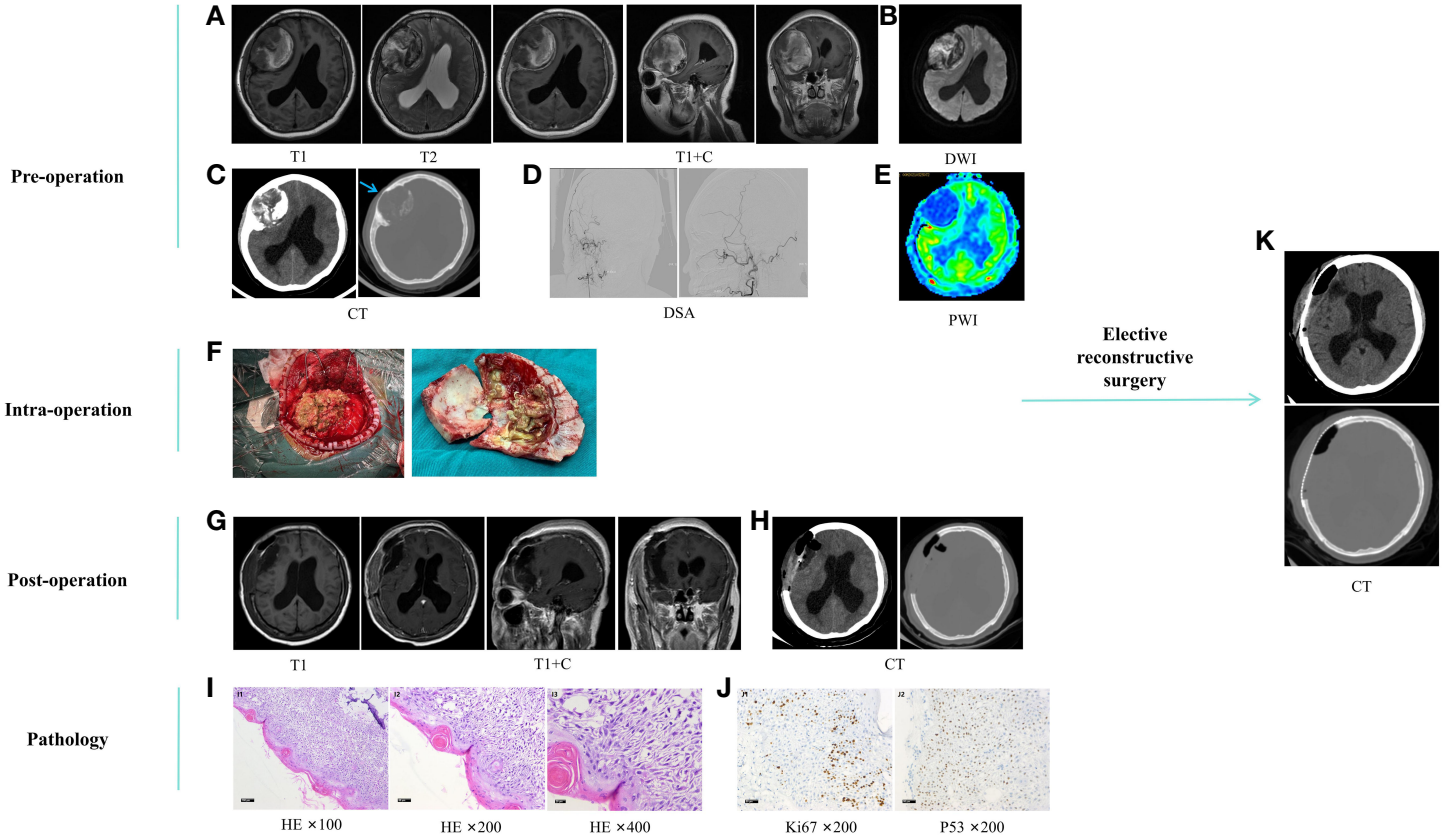
**(A–E)** preoperative imaging examination. Cranial MRI plain scan and enhanced **(A)**, DWI **(B)**, and CT **(C)** showed a space-occupying lesion in the skull at the right frontal-temporal-parietal roof (about 74.9*62.7*55.9mm) and showed hyperintensity on DWI, mixed with patchy hypointensity, fracture caused by tumor invasion of the skull (blue arrow), subfalcy herniation on the right side of the brain, mild obstructive hydrocephalus in the left lateral ventricle and the posterior lower part of the right lateral ventricle; In the anteroposterior and lateral positions of the right external carotid artery DSA **(D)**, no tumor supply artery was seen; Cranial PWI **(E)** shows hypoperfusion of the tumor. **(F)**, surgical process. **(G, H)**, postoperative imaging examination. Cranial MRI plain scan and enhanced **(G)** and CT **(H)** showed mass lesion resection combined with skull enlargement resection. **(I, J)**, postoperative pathological HE staining and immunohistochemistry, which reveals squamous epithelial cells covering the surface of the cystic wall, arranged in a star-like pattern, with a few wet keratinizations visible. No cellular atypia is observed, but there is minimal calcification in the stroma. I1-I3, HE ×100, ×200, ×400, gradually enlarged images of the same field of view. J1, Ki67 ×200, showing an approximately 20% increase in proliferation index; J2, P53 ×200, showing wild-type expression. **(K)**, scheduled craniotomy for repairing skull defects. MRI, Magnetic Resonance Imaging. DWI, Diffusion-Weighted Imaging; DSA, Digital Subtraction Angiography; PWI, Perfusion-Weighted Imaging; HE, Hematoxylin-Eosin.

On March 23, 2021, a 53-year-old female patient presented with a 20-day history of headaches and bilateral lower limb weakness for 10 days. Physical examination revealed slow and unsteady gait. An occupying lesion was observed in the right frontal-temporal-parietal region of the skull without supplying arteries on the Magnetic Resonance Imaging (MRI), Computed Tomography (CT), and Digital Subtraction Angiography (DSA) (**Figures 1A–E**). On March 25, 2021, a right cranial bone tumor margin expansion resection was performed. No tumor invasion was observed during surgery in the dura mater, which appeared dark blue. A subdural hematoma was observed after the dura mater was incised (**Figure 1F**). The patient’s motor functions recovered normally after surgery. Postoperative follow-up imaging examinations showed tumor resection (**Figures 1G, H**). Pathological examination after surgery revealed an AM of the cranial bone (**Figures 1I, J**). One month later, the patient underwent second-stage skull defect repair, and an appropriate amount of subcutaneous tissue was taken and sent to conventional pathology during the operation, and the results were not abnormal. Postoperative cranial CT showed that the skull reconstruction was good (**Figure 1K**), the head incision was well healed, there was no redness and swelling and exudation, and the sutures were removed and discharged.

**Figure 2:**
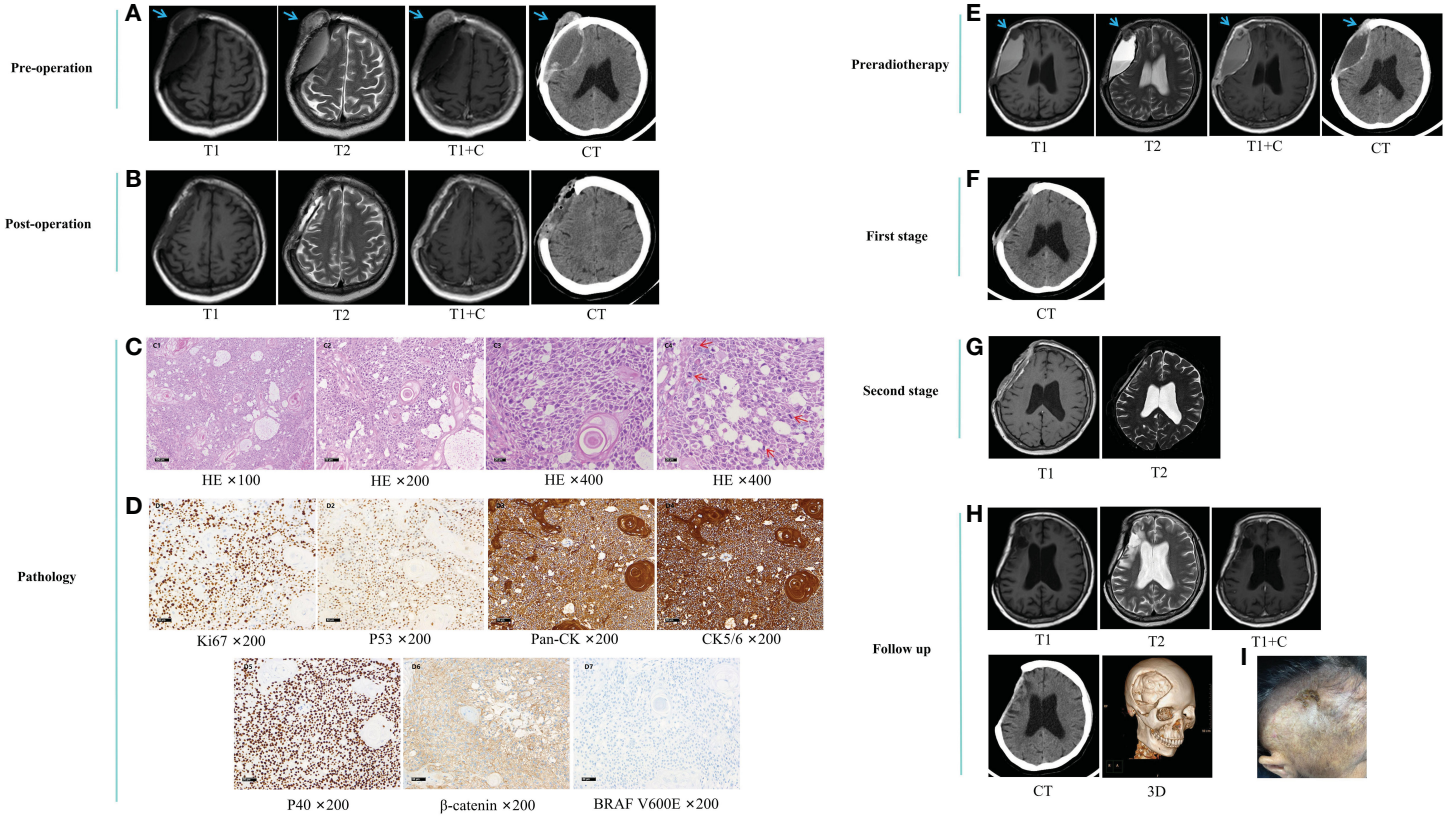
**(A)** preoperative imaging examination. **(B)** postoperative imaging examination. **(C)** postoperative pathological HE staining. Microscopic examination revealed diffuse infiltration of nests and starry arrangement of squamous epithelial cells. The epithelial cells were arranged in patches and nests, with locally loose background. Pearl formation was observed between the nests of squamous epithelial cells. The tumor cells exhibited obvious pleomorphism and frequent nuclear divisions. The tumor infiltrated the dura mater and surrounding soft tissues, with focal necrosis. C1-C3, HE ×100, ×200, and ×400 magnification of the same field, showing prominent nucleoli and significant cellular pleomorphism. C4, HE ×400, demonstrating increased nuclear divisions in other fields. **(D)**, postoperative immunohistochemical staining. D1, Ki67 ×200, showing an approximately 60% increase in proliferative index. D2, P53 ×200, showing wild-type expression. D3, Pan-CK ×200, positive. D4, CK5/6 ×200, positive. D5, P40 ×200, positive. D6, β-catenin ×200, cytoplasmic positive. D7, BRAF V600E ×200, negative. **(E)** pre-radiotherapy imaging examination. **(F)** imaging examination after the first phase. **(G)** imaging examination after the second phase. **(H, I)**, follow-up radiological examination and images after 10 months. Blue arrows indicate the tumor. Red arrows indicate nuclear divisions. HE, Hematoxylin-Eosin.

On August 3, 2022, imaging examinations showed tumor recurrence on the outer edge of a previous repair site in the right frontal lobe with a hematoma surrounding the surgical site (**Figure 2A**). The patient underwent resection of the right frontal lesion on August 11, and postoperative imaging examinations confirmed removal of the recurrent tumor (**Figure 2B**). Postoperative pathological analysis revealed malignant transformation of the AM, characterized by noticeable cellular abnormalities and increased cellular proliferation following recurrence (**Figures 2C, D**).

On October 12, 2022, the patient was admitted for stereotactic radiotherapy. Follow-up imaging examinations revealed a nodular enhancing lesion at the anterior edge of the surgical area (**Figure 2E**). After multidisciplinary discussions, it was determined that there was recurrence of cranial bone AC. Consequently, a three-stage localized boost radiotherapy plan was initiated, involving a prescription dose of 56 Gy/28 f to the planning target volume (PTV) and an additional boost of 60 Gy/20 f to the frontal region, administered over a four-week treatment period. Prior to radiotherapy, there was onset of perforation and exudation at the surgical incision site on the tumor surface of the frontal region. We performed daily wound dressing changes and administered antimicrobials. Subsequent follow-up imaging examinations revealed a progressive reduction in tumor size and fluid accumulation (**Figures 2F, G**).

On October 12, 2023, the patient was readmitted for follow-up examination. Follow-up imaging examinations demonstrated no evidence of tumor recurrence (**Figure 2H**) and metastasis. Examination revealed that the scalp incision overlying the original tumor in the frontal region had healed with the presence of a scab (**Figure 2I**).

### 2.2 Review of literature

Literature review identified five reported cases of primary cranial bone AM or AMCa between 1964 and 2022 (5–9) (**Table 1**). It revealed the diagnosis of both conditions can pose certain challenges owing to their rarity and overlapping presentation with other cranial tumors, such as osteoblastoma and cranial metastases. Two patients (one male and one female, with a mean age of 20 years) with primary temporal bone AM were managed with either surgical resection or radiation therapy alone; however, no follow-up evidence of recurrence and long-term outcomes are available. All the three patients (two females and one male, with a mean age of 43 years) with primary cranial base AMCa, experienced post-treatment recurrences. Two patients underwent surgical intervention combined with radiation therapy, resulting in one fatality, while one patient underwent surgical treatment alone and died 15 months postoperatively. Among the 3 cases of AMCa, we found that one case was malignantly transformed from a benign tumor lesion, and gradually began to have pathological manifestations with obvious cytological atypia and increased mitosis, which was similar to the evolution of our disease. The course of the remaining 2 cases may be considered primary. According to current reports, AMCa is more common from the primary, and secondary is relatively rare. In this report, we present the first documented case of malignant transformation of primary cranial bone (frontal-temporal-parietal regions) AM.

**Table 1:** Clinical Characteristics of Previously Reported Cases of Ameloblastoma (AM) or Ameloblastic Carcinoma (AMCa).

Year	country	Author	Sex	Age(years)	Pathology	Location	Surgery	Radiotherapy	BRAF V600E	Follow-up (months)	Recurrence	Outcome
1964	United Arab Republic	SAMY et al. (5)	F	23	AM	Temporal bone	–	+	*NA	*NA	*NA	No evidence of disease
2005	Turkey	Ozlugedik et al. (6)	F	23	AMCa	Anterior skull base	+	+	–	25	+	No evidence of disease
2010	China	Gao et al. (7)	F	42	AMCa	Intrasellar region	+	–	–	13	+	Patient died
2017	Croatia	Košec et al. (8)	M	17	AM	Temporal bone	+	–	*NA	7	Relatively similar to the past	No evidence of disease
2020	Croatia	Tarle et al. (9)	M	64	AMCa	Skull base	+	+	+	14	+	Patient died

*NA – not available; “+”, indicates the presence of surgical or radiotherapy treatment or the presence of a genetic mutation or a recurrence of the lesion. “–”, indicates no surgical or radiotherapy treatment or no genetic mutation or no recurrence of the lesion.

## 3 Discussion

We integrate all the information from the patient’s multiple hospital admissions and consider that the lesion is a malignant transformation from an AM growing from the skull. Actually ameloblast differentiated from inner dental epithelium which is originate from enamel organ during the bell stage of the tooth development, and the diagnosis is based on morphology (1, 2). While AM commonly occurs in the jawbones, its occurrence in cranial bones is extremely uncommon. AM is a benign tumor with local aggressiveness, and the biological behavior is characterized by local recurrence in the same site. With each recurrence, the degree of cellular malignancy increases, leading to pleomorphism and atypia (1, 2). According to the latest World Health Organization (WHO) classification of odontogenic tumors in 2022, AMCa is defined as a rare odontogenic malignancy that combines the histologic features of AM with cytological atypia (1). The 5-year survival rate is reported to be 69.1%, whereas patients with metastasis have only 21.4% (1, 10). Patients with AMCa exhibit more aggressive clinical symptoms than those with AM. Furthermore, it differs from AM in terms of its rapid growth, cortical perforation, pain, and sensory abnormalities (11). AMCa usually develops *de novo*, but it can also occur in benign ameloblastoma that has been long-existing, untreated, or recurred (11). Histologically, AMCa is similar to AM in that it mainly presents as a stellate reticular central epithelium and surrounding basal palisade cells, with opposite nuclear polarity; However, AMCa also has obvious malignant features, such as cellular atypia, nuclear hyperchromatic, and increased mitotic activity (12, 13). The difficulty in diagnosing AMCa lies in the lack of uniform criteria for cellular atypia, and the limited tissue samples examined can easily lead to misdiagnosis or missed diagnosis. Immunohistochemical staining can help distinguish AMCa from AM, such as Ki-67 marker index and p53 expression (9, 14). Malignant transformation of AM may be closely related to a long medical history, chronic inflammation after surgery, multiple surgeries, radiation therapy, and chemotherapy, while secondary malignant transformation usually follows benign AM (15). However, the mechanism of malignant transformation is currently unknown.

In this case, the first postoperative pathology showed AM, and the pathology showed that the cell morphology was mild and it was a typical enamelogenic epithelium (**Figures 1-I1**). The second pathological result showed the malignant transformation of AM, which was obvious cell atypia and active proliferation after recurrence and progression (**Figures 2-C1, 2-C4**). Immunohistochemical staining of Ki-67 proliferation markers showed that the Ki-67 marker index was approximately 20% and 60% in benign and cancerous lesions, respectively (**Figures 1-J1**, **2-D1**). Compared with previous cases (14), the Ki-67 index in this case was higher, suggesting that the tumor had high proliferative potential and high malignancy. In this case, the patient had a history of AM at the same site, and the last resected specimen had a malignant component. Therefore, we consider the malignant transformation from AM to AMCa, which is more consistent with the extremely rare secondary form of AMCa.

AMCa is clinically similar to AM (12–16). When in the oral area, it usually manifests as swelling, followed by pain, ulcers, paresthesias, and trismus. When in the cranial region, whether primary or secondary, it can present with findings associated with intracranial neoplastic lesions. However, due to the extremely complex anatomy of the jaw region, most patients will still have diseased tissue after radical surgery, which leads to a high recurrence rate. AMCa can be more aggressive than most typical AMs. Both may have distant metastases, most commonly in the lungs, but bone, liver, or brain metastases have also been reported (3, 12, 13). These tumors are prone to multiple recurrences and require long-term follow-up.

While biopsy is the gold standard for the diagnosis of AM and AMCa, advanced imaging technology plays an important role in their diagnosis, treatment planning, and monitoring. The imaging features of AMCa are similar to those of AM (3, 12). Imaging evaluation of both is usually done using plain x-rays, CT, MRI, and positron emission tomography (PET-CT). Among them, CT is considered to be the most useful diagnostic imaging modality, MRI provides more complete information about soft tissue and bone marrow and extraosseous invasions, and PET-CT is mainly used to detect distant metastases of tumors (3, 12). Neither has typical imaging features; Therefore, biopsy remains key to confirm the diagnosis. Biopsy and imaging can also be used to help distinguish between the differential diagnosis of adamantinomatous craniopharyngioma, ossifying fibroma, osteomyelitis, giant cell tumor, cystic fibrodysplasia, myeloma, and sarcoma (12).

At present, various chemotherapy drugs have been reported to be useful, but the treatment effect is not ideal; When in the case of metastases, chemotherapy remains the only treatment option (12). Radiation therapy is effective in some cases for AM and AMCa treatment, and it can be used in patients with microscopic or macroscopic residual lesions after surgery, recurrence after surgery, or disease that is not amenable to resection (12, 17, 18). As a result, newer radiotherapy techniques such as image-guided radiotherapy, stereotactic radiotherapy, intensity-modulated radiotherapy, and proton beam therapy may become new treatment options (12).

Chemotherapy and radiotherapy are limited in the treatment of ameloblastoma, surgery is still the mainstay of treatment, and molecularly targeted therapy is the new adjuvant treatment. Currently, there are no standardized treatment protocols for AM or AMCa. The combined treatment approaches often include aggressive surgical resection, radiation therapy, or chemotherapy. Interestingly, the high recurrence rate of AM or AMCa is not solely determined by tumor size or histological type but is primarily attributed to inadequate local excision. Once the lesion invades the adjacent soft tissues, the recurrence rate increases owing to challenges in identifying the tumor boundaries within the soft tissue, making curative surgery extremely difficult and resulting in higher rates of postoperative recurrence. Therefore, preoperative MRI and CT scans, intraoperative assessment of soft tissue margins through frozen sections, and the use of imaging techniques play significant roles in the comprehensive management of these lesions. For AM, a recommended resection margin of 1.5-2 cm beyond the radiographic bone margin is advised, whereas for AMCa cases, a clearance of 2-3 cm of the trabecular bone margin is recommended (12, 16, 19). Therefore, relevant studies have found that there is still a certain recurrence rate after surgery, regardless of surgery alone or combined with chemoradiotherapy. In this regard, experts have begun to gradually explore the research of molecular targeted therapy (20–23).

Molecularly targeted therapy research is focused on BRAF mutations, as 63% to 82% of AM patients and 38% of AMCa patients have BRAF V600E mutations (9, 20–24).And most of them are located in the mandible and are sensitive to targeted drugs such as vemurafenib. BRAF-V600E mutations have also been found to be associated with aggressive behavior in AMCa, which can be accurately diagnosed by immunohistochemistry, which can be used to assess prognosis and select treatment (12, 15, 24–27). A small number of studies have found that SMO-mutated AM are predominantly located in the maxilla, however, more clinical studies are needed to overcome resistance to targeted drugs and reduce adverse effects (12, 24, 28).

Only a small number of AM have been found to contain p53 mutations, and they have also been found to be visible in AMCa, which may be associated with malignant transformation, which provides new ideas for the development of novel therapeutic agents (14, 24). Postoperative immunohistochemistry showed BRAF-V600E negative and p53 wild-type expression. This may suggest that mutations in the AM and AMCa genes are associated with the location of the disease. Therefore, further research on the mechanism of action of new molecularly targeted therapy drugs and explore new treatment strategies can provide better options for individualized treatment of patients with AM or AMCa.

## 4 Conclusion

Primary cranial bone AM or AMCa are rare tumors of skull. The mechanism underlying the malignant transformation of AM into AMCa is still not fully understood. Both conditions exhibit high recurrence rates and demonstrate a highly invasive nature. In summary, the treatment of these conditions requires the implementation of the most aggressive treatment methods and close neuro-radiological follow-up, resembling the approach we take when managing malignant pathologies.

## Data availability statement

The original contributions presented in the study are included in the article/supplementary material. Further inquiries can be directed to the corresponding authors.

## Ethics statement

The studies involving humans were approved by Guangdong Sanjiu Brain Hospital. The studies were conducted in accordance with the local legislation and institutional requirements. The participants provided their written informed consent to participate in this study. Written informed consent was obtained from the individual(s) for the publication of any potentially identifiable images or data included in this article.

## Author contributions

HX: Writing – original draft, Writing – review & editing. JT: Writing – original draft, Writing – review & editing. DF: Writing – original draft, Writing – review & editing.
